# Reduced Neutrophil‐to‐Eosinophil Ratio in Chronic Dacryocystitis: A Hematological Clue to Allergic Pathogenesis

**DOI:** 10.1155/joph/8447280

**Published:** 2026-06-27

**Authors:** Gokhan Ozgur, Konuralp Yakar, Onur Gokmen

**Affiliations:** ^1^ Department of Ophthalmology, Medicana International Samsun Hospital, Samsun, Turkey; ^2^ Department of Ophthalmology, Faculty of Medicine, Atılım University, Ankara, Turkey, atilim.edu.tr; ^3^ Department of Ophthalmology, Faculty of Medicine, Yuzuncu Yıl University, Van, Turkey, yyu.edu.tr

**Keywords:** dacryocystitis, monocyte-to-lymphocyte, neutrophil-to-eosinophil, neutrophil-to-lymphocyte, neutrophil-to-monocyte, platelet-to-lymphocyte

## Abstract

**Aim:**

To examine complete blood count parameters for differences in chronic dacryocystitis compared to controls, exploring their potential link to a possible allergic‐inflammatory contribution in lacrimal surgery.

**Methods:**

This retrospective study included 190 adults aged 18–83 years who underwent external dacryocystorhinostomy (DCR) for primary acquired nasolacrimal duct obstruction (PANDO) with chronic dacryocystitis between January 2018 and June 2023. Preoperative peripheral blood samples were analyzed for leukocyte, neutrophil, platelet, eosinophil, and monocyte counts. Neutrophil‐to‐lymphocyte ratio (NLR), platelet‐to‐lymphocyte ratio (PLR), neutrophil‐to‐monocyte ratio (NMR), monocyte‐to‐lymphocyte ratio (MLR), and neutrophil‐to‐eosinophil ratio (NER) were calculated and compared with a control group consisting of age‐ and sex‐matched healthy individuals who underwent routine ophthalmological examinations.

**Results:**

Eosinophil counts were significantly higher (*p* = 0.003) and NER significantly lower (*p* = 0.014) in the DCR group compared to controls, with no significant differences in other parameters (*p* > 0.05).

**Conclusion:**

Elevated eosinophil levels and reduced NER suggest a possible allergic‐inflammatory contribution to chronic dacryocystitis. NER may serve as a potential biomarker for identifying patients with an allergic‐inflammatory profile; however, prospective studies are required to establish its clinical utility.

## 1. Introduction

Primary acquired nasolacrimal duct obstruction (PANDO) represents a common condition in adults, primarily affecting middle‐aged women and typically presenting with unilateral epiphora. This symptom can be linked to acute or chronic infections, often termed dacryocystitis, which may occur alongside the obstruction [1]. Chronic dacryocystitis arises as an infectious process following nasolacrimal duct narrowing or blockage, with lacrimal discharge being the most prominent sign. Patients often exhibit conjunctival redness at the inner canthus and may express mucoid or purulent material from the lacrimal puncta when the affected area is compressed. The condition is frequently driven by Gram‐positive bacteria, notably *Staphylococcus aureus*, potentially leading to serious complications such as keratitis or orbital cellulitis [2, 3]. Factors such as rhinitis, enlarged inferior turbinates, deviated nasal septum, nasal polyps, and trauma to the lacrimal duct are commonly implicated in its development [4]. For chronic dacryocystitis associated with PANDO, the preferred treatments are external or endonasal dacryocystorhinostomy (DCR) [5].

Systemic inflammation can be evaluated using various biochemical assays, though some of these methods remain expensive and intricate. Recently, complete blood count (CBC)–derived inflammatory indices—including the neutrophil‐to‐lymphocyte ratio (NLR), platelet‐to‐lymphocyte ratio (PLR), monocyte‐to‐lymphocyte ratio (MLR), neutrophil‐to‐monocyte ratio (NMR), and neutrophil‐to‐eosinophil ratio (NER)—have gained increasing attention as inexpensive and readily available biomarkers reflecting systemic inflammatory and immune responses. These parameters have been extensively investigated across many systemic and inflammatory diseases, such as cardiovascular, neurological, oncological, autoimmune, and chronic inflammatory disorders, where they have demonstrated potential diagnostic and prognostic value as accessible indicators of immune dysregulation [6–8]. Recent studies have further highlighted the clinical relevance of these hematological markers in inflammatory and immune‐mediated conditions, supporting their role as surrogate indicators of chronic inflammation and tissue remodeling [9–11].

The objective of this study is to investigate differences in these indices between patients with chronic dacryocystitis and healthy controls, hypothesizing that alterations, particularly a reduced NER, may reflect a possible allergic contribution to nasolacrimal duct obstruction. While studies have explored microbiological profiles and histopathological changes in chronic dacryocystitis, there is a notable lack of research specifically examining these hematological inflammatory indices in this condition, representing a gap in the literature that this study seeks to address.

## 2. Materials and Methods

The blood test results of 190 adults aged between 18 and 83 years who underwent external DCR surgery for the treatment of chronic dacryocystitis due to PANDO at our ophthalmology clinic between January 2018 and June 2023 were included in the study. A control group of 190 healthy individuals was selected to match the DCR group in terms of age, sex, and sample size to ensure comparability of hematological parameters and minimize confounding factors such as demographic differences. The research was approved by the local ethics committee (date: 06.09.2023, number: 2023/16/15) and conducted in accordance with the principles of the Declaration of Helsinki. Informed written consent was obtained from all participants prior to the procedure.

All participants underwent a comprehensive ophthalmological examination, including visual acuity assessment, biomicroscopy, and fundus evaluations, examinations of the lower and upper canaliculi, and inspection and palpation of the lacrimal sac. Ocular surface evaluation (e.g., tear breakup time and Schirmer test) was not routinely performed unless clinically indicated. Chronic dacryocystitis was defined as persistent symptoms, including epiphora and mucoid or purulent discharge, lasting for at least 3 months, confirmed by clinical evaluation and nasolacrimal lavage demonstrating obstruction. Nasolacrimal lavage was performed to assess nasolacrimal duct patency, and chronic dacryocystitis secondary to PANDO was diagnosed in patients who did not report saline passage into the nasopharynx during irrigation, indicating complete obstruction. Patients with a history of acute dacryocystitis were included only if the acute episode had resolved at least 4 weeks prior to surgery, ensuring the chronic nature of the condition. Due to the retrospective design, patient data were retrieved from medical records. A total of 225 patients diagnosed with PANDO were initially screened; however, 35 patients were excluded for not meeting the specific inclusion and exclusion criteria or due to missing laboratory data. Consequently, 190 patients who met all requirements were included in the final analysis. Exclusion criteria for the DCR group, identified through chart review, included systemic chronic diseases (e.g., diabetes, cardiovascular disease, cancer, and autoimmune disorders), pregnancy, use of oral contraceptives, steroids, or immunosuppressive medications, smoking, active ocular or systemic infection, unresolved acute dacryocystitis within 4 weeks of surgery, suspected nasal pathology (e.g., rhinitis and nasal polyps) requiring ENT consultation, and intraoperative or postoperative complications (e.g., excessive bleeding and infection). While patients with overt and documented active rhinitis or major systemic atopic conditions were excluded based on available clinical records, a definitive exclusion of all individuals with remote or subclinical allergic histories was limited by the retrospective design. For all patients, CBC results obtained within 7 days prior to external DCR surgery were included in the study, as identified through retrospective chart review. Blood samples collected earlier than 7 days preoperatively were excluded. The healthy control group was recruited from patients who presented to our outpatient ophthalmology clinic for routine checkups or refractive errors during the same study period. The control group comprised healthy male and female participants without known systemic chronic diseases, such as diabetes, cardiovascular disease, cancer, or autoimmune disorders, who were not pregnant, did not use oral contraceptives, steroids, or other immunosuppressive or immunomodulatory medications, were nonsmokers, exhibited no signs of ocular or systemic infection, had no ocular pathology and demonstrated complete patency of the nasolacrimal passage, as confirmed by a nasolacrimal lavage examination.

Blood samples were obtained from the antecubital region following overnight fasting, placed into CBC tubes, and analyzed using an automated cell counter (Sysmex XN‐1000, Sysmex Corporation, Kobe, Japan). Leukocyte count, neutrophil count, platelet count, eosinophil count, and monocyte count data were extracted from the blood test results. Subsequently, the NLR, PLR, NMR, MLR, and NER were calculated.

### 2.1. Statistical Analysis

Statistical analyses were conducted utilizing IBM Statistical Package for the Social Sciences (SPSS) Statistics for Windows (Version 21.0. Armonk, NY: IBM Corp.). Normality of the data was assessed using Kolmogorov–Smirnov and Shapiro–Wilk tests. Since the variables were found to follow a nonnormal distribution, the Mann–Whitney *U* test was used for all intergroup comparisons. Continuous variables are presented as median and interquartile range (IQR) (25th–75th percentiles), while categorical variables are expressed as numbers and percentages. To assess the magnitude of the differences between the groups, effect sizes were calculated using the rank‐biserial correlation coefficient (r), where values of 0.10, 0.30, and 0.50 were considered to represent small, medium, and large effects, respectively. *p* values less than 0.05 were considered statistically significant.

## 3. Results

A total of 190 patients who underwent external DCR surgery for chronic dacryocystitis due to PANDO were included in the study, along with 190 age‐ and sex‐matched controls. The mean age of the DCR group was 54.97 ± 15.4 years (range: 18–83) and that of the control group was 52.07 ± 18.7 years (range: 21–82). The groups were similar in terms of age and sex distribution. Both groups consisted of 124 (65%) female and 66 (35%) male participants.

The levels of inflammatory blood cell parameters in the chronic dacryocystitis and control groups are shown in Table 1. There were no significant differences between the chronic dacryocystitis and control groups in lymphocytes, neutrophil, monocyte, and platelet counts, but eosinophil count was significantly higher in the chronic dacryocystitis group (*p* = 0.003 with a small‐to‐medium effect size of *r* = 0.21) (Table 1).

**TABLE 1:** Comparison of blood parameters between the chronic dacryocystitis and control group.

Parameter	Chronic dacryocystitis (*n* = 190)	Control group (*n* = 190)	*p* value^∗^	Effect size (*r*)^∗∗^
Neutrophils (× 10^9^/L)	3.51 (2.75–4.54)	3.68 (2.81–4.79)	0.384	—
Lymphocytes (× 10^9^/L)	2.01 (1.63–2.50)	1.94 (1.59–2.45)	0.428	—
Platelet (× 10^9^/L)	255 (206–301)	258 (209–314)	0.714	—
Monocyte (× 10^9^/L)	0.46 (0.35–0.59)	0.48 (0.36–0.61)	0.521	—
Eosinophil (× 10^9^/L)	0.12 (0.07–0.19)	0.09 (0.05–0.14)	**0.003**	**0.21**

*Note:* Data are expressed as median and interquartile range (IQR) [25th–75th percentiles]. Effect sizes are reported only for statistically significant comparisons, *p* values < 0.05 were considered statistically significant.

^∗^Mann–Whitney *U* test was used for all comparisons.

^∗∗^Effect size was calculated using the rank‐biserial correlation coefficient (*r*).

A comparison of the NLR, PLR, NMR, and MLR values between the groups revealed no significant differences (*p* > 0.05), whereas the NER was significantly lower in the chronic dacryocystitis group (*p* = 0.014 demonstrating a corresponding effect size of *r* = 0.19) (Table 2).

**TABLE 2:** Comparison of NLR, PLR, NMR, MLR, and NER between chronic dacryocystitis and the control group.

Parameter	Chronic dacryocystitis (*n* = 190)	Control group (*n* = 190)	*p* value^∗^	Effect size (*r*)^∗∗^
NLR	1.72 (1.26–2.41)	1.88 (1.31–2.58)	0.142	—
PLR	120.4 (95.1–154.2)	122.7 (98.3–161.5)	0.744	—
NMR	7.84 (6.12–9.75)	8.41 (6.38–10.42)	0.126	—
MLR	0.22 (0.17–0.30)	0.23 (0.18–0.32)	0.322	—
NER	28.5 (17.4–46.2)	39.8 (24.5–62.1)	**0.014**	**0.19**

*Note:* Data are expressed as median and interquartile range (IQR) [25th–75th percentiles] (effect sizes are reported only for statistically significant comparisons), *p* values < 0.05 were considered statistically significant.

Abbreviations: MLR, monocyte‐to‐lymphocyte ratio; NER, neutrophil‐to‐eosinophil ratio; NLR, neutrophil‐to‐lymphocyte ratio; NMR, neutrophil‐to‐monocyte ratio; PLR, platelet‐to‐lymphocyte ratio.

^∗^Mann–Whitney *U* test was used for all comparisons.

^∗∗^Effect size was calculated using the rank‐biserial correlation coefficient (*r*).

## 4. Discussion

Chronic dacryocystitis remains a cornerstone of lacrimal surgery within ophthalmology, with external DCR established as the gold standard for restoring drainage in PANDO [12, 13]. To our knowledge, this study is among the first to investigate hematological inflammatory indices, particularly the NER, in chronic dacryocystitis, offering a novel perspective on a possible allergic‐inflammatory contribution to its pathogenesis. Our investigation revealed a statistically significant increase in eosinophil levels (*p* = 0.003) and a reduction in the NER (*p* = 0.014) in 190 patients with chronic dacryocystitis. These findings challenge the traditional infection‐centered paradigm, suggesting that allergic‐inflammatory mechanisms may contribute to the pathogenesis of lacrimal sac obstruction. This finding aligns with the growing body of evidence linking mucosal hypersensitivity to nasolacrimal pathology, an area gaining prominence in ophthalmic practice [14]. In our clinic, all external DCR procedures are performed under general anesthesia, and a CBC is routinely obtained preoperatively to assess baseline hematological status and rule out systemic infection or inflammation, ensuring patient safety and guiding perioperative management.

Eosinophils, which play a crucial role in allergic inflammation, may induce mucosal edema, fibrosis, or epithelial remodeling in the lacrimal sac, potentially predisposing it to obstruction and subsequent bacterial overgrowth [15, 16]. Mast cells play a central role in the acute phase of allergic inflammation through degranulation and release of histamine and other mediators, which can promote eosinophil recruitment [17]. In a chronic setting, eosinophils become the dominant cell type and contribute to tissue remodeling and fibrosis [18]. Although direct mast cell or eosinophil infiltration in lacrimal sac tissue was not evaluated in this retrospective study, our finding of elevated eosinophil counts and reduced NER supports the presence of a chronic eosinophil‐dominant inflammatory process. Antihistamines may have a potential therapeutic role by blocking histamine‐mediated mucosal edema and vascular permeability in the nasolacrimal drainage system. Preoperative management of allergic rhinitis with antihistamines and intranasal corticosteroids has been shown to improve DCR success rates and reduce postoperative complications [19]. This approach may help mitigate the allergic component that contributes to fibrosis and obstruction in PANDO. The absence of systemic inflammatory markers, such as NLR or PLR, suggests a localized process that may prevent detection in routine ophthalmic evaluations [14, 20]. However, as highlighted in contemporary literature, these novel CBC‐derived ratios serve as highly accessible, low‐cost, and noninvasive biomarkers capable of reflecting broader pathways of immune dysregulation, chronic inflammation, and systemic cellular responses [9–11]. While these indices have established diagnostic and prognostic value across diverse oncological, systemic, and chronic inflammatory conditions, our study contextualizes their potential utility within a localized ocular pathology, suggesting that a subclinical or localized allergic‐inflammatory involvement might be reflected through subtle alterations in peripheral blood parameters like the NER.

The ophthalmological literature provides a robust foundation for this hypothesis. Patients with PANDO frequently exhibit allergic rhinitis or chronic rhinosinusitis, conditions linked to mucosal inflammation and associated with poorer DCR outcomes [21, 22]. Histopathological analyses of lacrimal sacs in chronic dacryocystitis have identified chronic inflammation characterized by lymphocytic infiltration and epithelial changes, pointing to an underlying inflammatory process [22, 23]. Research by Koturović et al. [24] has emphasized the clinical relevance of routine lacrimal sac biopsies during DCR, while Ali et al. [25] have highlighted mucosal remodeling in PANDO, supporting a multifactorial etiology that extends beyond mechanical obstruction alone. Our data build on these observations, suggesting that a reduced NER reflects an allergic component mediated by inflammation, offering ophthalmologists a hematological perspective to enhance lacrimal surgery outcomes.

From a clinical perspective, a reduced NER may indicate an allergic predisposition, guiding ophthalmologists toward adjunctive therapies such as antihistamines, intranasal corticosteroids, or allergy testing prior to lacrimal surgery. Such interventions could enhance lacrimal drainage restoration and reduce recurrence, a persistent challenge in lacrimal surgery. Allergic rhinitis has been demonstrated to compromise DCR success, with higher failure rates observed in patients with untreated nasal allergies [19]. Tugrul et al. [19] established that the preoperative management of allergic rhinitis improved DCR outcomes and reduced postoperative complications, whereas Sevimli et al. [20] observed alterations in inflammatory markers post‐DCR, suggesting that surgery modulates local inflammation. Recent studies by Eriman et al. [26] and Lee et al. [27] further correlated nasal pathology with DCR failure, advocating preoperative nasal assessment. Moreover, eosinophil‐targeted therapies, increasingly investigated in allergic diseases, might have a role in refractory cases.

Beyond surgical outcomes, the prognostic potential of NER warrants consideration. Systemic eosinophilia has been associated with inflammatory ocular conditions such as vernal keratoconjunctivitis [28], and local eosinophil activity is implicated in nasal polyposis, a known risk factor for nasolacrimal obstruction [29, 30]. Our observation of reduced NER, potentially indicative of relative eosinophil elevation, could serve as a link between systemic allergy and localized lacrimal pathology, aligning with the precision medicine paradigm in ophthalmology.

This study has several limitations that should be acknowledged. Most notably, given our central hypothesis of an allergic pathogenesis, the inability to assess serum IgE levels, the lack of histopathological confirmation of eosinophilic tissue infiltration in the lacrimal sac due to the absence of mucosal biopsies, and the lack of formal allergy testing (such as skin prick tests) must be emphasized as major limitations of this research. Furthermore, although patients with overt nasal pathologies were excluded, the retrospective nature of the study prevented a detailed grading of atopic symptoms or a comprehensive assessment of personal and family allergic histories using standardized screening tools. Consequently, the potential confounding effect of unrecorded subclinical atopic conditions on baseline eosinophil levels and novel hematological indices cannot be entirely ruled out. Although we excluded patients with acute dacryocystitis within 4 weeks prior to surgery to minimize confounding, the potential residual effects of prior systemic antibiotic or analgesic use could not be completely ruled out. Moreover, inconsistent documentation regarding the frequency and timing of previous acute episodes hindered assessment of their cumulative influence on hematologic parameters. The retrospective nature of the study also resulted in incomplete follow‐up data, preventing the evaluation of postoperative outcomes or serial hematologic trends. Furthermore, the lack of routine ocular surface assessments—such as tear breakup time or Schirmer testing—restricted our understanding of potential associations between systemic inflammation and ocular surface status [31]. While elevated eosinophil counts can result from nonallergic causes including parasitic infections, tuberculosis, or certain medications, we attempted to mitigate this by excluding patients with systemic infections or recent drug use [32]. Nevertheless, the retrospective design limited our ability to comprehensively screen for parasitic diseases. Despite these limitations, the observed elevation in eosinophil counts and reduction in NERs support a potential allergic contribution to chronic dacryocystitis, underscoring the need for future prospective studies to clarify these findings.

## 5. Conclusion

Increased eosinophil levels and reduced NER in patients with chronic dacryocystitis suggest a possible allergic‐inflammatory contribution to the pathogenesis of this condition. Assessment of NER prior to lacrimal surgery may help identify patients with a potential allergic‐inflammatory profile. However, prospective studies incorporating allergy testing, histopathological evaluation, and targeted antiallergic interventions are needed to determine the clinical utility of NER and to establish whether such interventions improve DCR outcomes. Further studies correlating NER with lacrimal sac biopsy findings, eosinophil counts, and allergy profiles may help clarify the underlying mechanisms of chronic dacryocystitis.

## Author Contributions

Significant contribution to conception and design: Gokhan Ozgur, Onur Gokmen, and Konuralp Yakar.

Data Acquisition: Gokhan Ozgur.

Data analysis and interpretation: Onur Gokmen, Konuralp Yakar, and Gokhan Ozgur.

Manuscript drafting: Konuralp Yakar, Onur Gokmen, and Gokhan Ozgur.

Significant intellectual content revision of the manuscript: Konuralp Yakar and Gokhan Ozgur.

Statistical analysis: Onur Gokmen and Konuralp Yakar.

Research group leadership: Gokhan Ozgur.

## Funding

No funding or sponsorship was received for this study or publication of this article.

## Disclosure

All named authors meet the International Committee of Medical Journal Editors (ICMJE) criteria for authorship of this manuscript, take responsibility for the integrity of the work as a whole, and have given final approval for the version to be published.

## Ethics Statement

The research was approved by the local ethics committee (date: 06.09.2023, number: 2023/16/15) and conducted in accordance with the principles of the Declaration of Helsinki. Informed written consent was obtained from all participants prior to the procedure.

## Consent

Please see the Ethics Statement.

## Conflicts of Interest

The authors declare no conflicts of interest.

## Data Availability

The datasets used and/or analyzed during the current study are available from the corresponding author upon reasonable request.
